# Special Issue: Microscopy Methods for Virus Research

**DOI:** 10.3390/v18050551

**Published:** 2026-05-12

**Authors:** Krishanu Ray

**Affiliations:** 1 Division of Vaccine Research, Institute of Human Virology, University of Maryland School of Medicine, 725 West Lombard Street, Baltimore, MD 21201, USA; kray@som.umaryland.edu; 2 Department of Biochemistry and Molecular Biology, University of Maryland School of Medicine, 725 West Lombard Street, Baltimore, MD 21201, USA

Significant advances in microscopy methods for biomedical applications have been made in the past two decades, and these methods are now being actively pursued for virus research. Implementing quantitative advanced microscopic methods, including optical and electron microscopy imaging, can provide important information regarding the mechanism and dynamics of viral infection and identify targets for therapeutic intervention. These methods are opening new avenues for research in microbiology, particularly with respect to viruses. Furthermore, improvements in microscopy methods have allowed researchers to directly examine single viruses in their natural forms, generating new insights into their structure, function, and mechanism of action over time and/or in three dimensions. Specifically, recent progress in several super-resolved fluorescence microscopy, single molecule, and in vivo optical imaging methods, as well as novel fluorescence tags and electron microscopy methods including cryoEM and cryoET, opens the possibility of tracking viruses in host–pathogen interactions and evaluating dynamics at the molecular level. This is fundamental to providing unprecedented information for the development of interventions and therapeutic strategies. This Special Issue of *Viruses* brings together seven original research articles, two reviews and one CryoVirus data descriptor, highlighting the progress of imaging in the broad area of virus research including HIV-1 and SARS-CoV-2.

The contributions are diverse in scope: one review discussed RNA viral infections with multiscale microscopic methods, while the other review provided a snapshot of cryoEM for bridging the structural gap for the underrepresented viruses. One research article reported a flow cytometry-based application towards quantification of a viral infection. A solution-based quantitative fluorescence correlation spectroscopy approach conducted in-depth analyses on the differences between the antigenic properties of the soluble SARS-CoV-2 spike proteins versus the virion-associated envelope spikes. This study underscores the importance of studying viruses in their native, virion-associated states. A report on the multiplex microscopy assay evaluated the functioning of therapeutic and serum antibodies against emerging pathogens. Another article on quantitative fluorescence microscopy reported on the interactions between hantavirus nucleoprotein and glycoproteins. Additionally, the imaging of retroviral RNA genome heterodimers was achieved by applying the fluorescence complementation method. The application of super-resolution microscopy showed the responses of nuclear pores to mechanical stress. Finally, the use of expansion microscopy provided a new lens for studying HIV-1–cell interactions.

Briefly, the contributions in this Special Issue underscore that the future of virology is inherently multi-modal. Moreover, these articles point out that the integration of high-resolution imaging and quantitative analysis is not just an evolution of methods, but an important step forward in our ability to visualize and combat viral threats.

